# What Is the Simillimum?

**Published:** 1890-03

**Authors:** M. R. R. Wolff


### WHAT IS THE SIMILLIMUM?

(Addendum to the case in the January number.)

M. R. R. Wolff, M. D.

February 1st the patient writes: "For two or three days previous to appearance of menses I have a rushing, gurgling sound in my abdomen, sounding like water going through a syringe. When in bed, with no bands or belts touching me, it is the same, and does not seem to be gas, nor pass off any unusual amount."

Feb. 2d.—"Since writing the above have had my period of suffering. Menses, which were going on so well, stopped for four hours, causing much pain, but, coming on in a gush, relieved for a time, only to stop again for twelve hours, making me unable to sleep, and suffering very much until another big gush, when I was relieved instantly, and have suffered no more since, although still having menses a little. During the last stoppage there was for six hours a perfectly hard round-shaped ball of the neck of the womb, instead of a long shape, and immediately after the flow it resumed its natural shape, but with a soft, flabby feeling. There is no coming and going of the pains, but a steady, crowding, bursting feeling which extends all through the back and sides, and low down in the pelvis. Every time I have my menses, about four or five days previous, I have one or more pimples come on my chin, which fester a little and look very ugly to me. I have had fever, or flashes of fever, sometimes during half the day or night for several days, but no chilly sensations. I feel it in hands and feet and eyes."

Father and father's family, "salt rheum" people; the father in high degree. How treated I cannot say, but allopathically. (So, too, the suffering lady in childhood.) Mother's family, phthisical people, all dying early. It is Homœopathy which, so far, has saved the old lady.

Prescription.—One powder Psorin.$^{1500}$ (the highest I have), take at once, dry. The day next menses are expected take, in advance, with eight hours interval, one powder Mag-phos.$^{200}$ (the highest I have), three times. Should menses come on before these powders are taken, do not take more before pains are felt. Then one powder, and do not repeat except in case of relapse. (See proving of Mag-phos. in *Advance*, and compare pathogenesis of Mag-mur.)

Once more. What is the simillimum?

